# Clinical, randomized, double blind clinical trial to study the effect of parenteral supplementation with fish oil emulsion in the nutritional support in esophagectomized patients

**DOI:** 10.1097/MD.0000000000026426

**Published:** 2021-06-25

**Authors:** Ana Suárez-Lledó, Elisabet Leiva-Badosa, Josep M. Llop-Talaveron, Mónica Fernández-Alvarez, Leandre Farran-Teixidor, Mónica Miró-Martín, Nuria Virgili-Casas, Glòria Creus-Costas, Jordi Bas-Minguet, Elisabet Poyatos-Canton, Sergio Navarro-Velazquez, María B. Badia-Tahull

**Affiliations:** a Pharmacy Department; b General and Digestive Surgery Department; c Endocrinology and Nutrition Department; d Inmunology Laboratory, Bellvitge Hospital, University of Barcelona-IDIBELL, L’Hospitalet de Llobregat, Barcelona, Spain.

**Keywords:** anti-inflammatory, esophageal cancer, esophagectomy, fish-oil, immunomodulation, omega-3

## Abstract

**Introduction:**

Esophagectomy is a major surgery with a high degree of catabolic and post-surgical inflammatory response accompanied by high morbidity and significant mortality. Post-surgical nutritional support via enteral administration of ω-3 fatty acids has been seen to be effective although its bad tolerance. There are few clinical trials with parenteral ω-3 fatty acids in these patients. We propose to investigate the effect of combining a parenteral fish oil lipid emulsion with the standard enteral nutrition (EN) support.

**Materials and methods:**

Prospective, single-center, randomized, double-blind study in esophagectomized patients, and treated after surgery with parenteral lipid emulsions of ω-3 fatty acids or a mixture of ω-6 long-chain triglycerides/short-chain triglycerides 50%. These emulsions will be added to the standard nutritional support in continuous infusion until 5 days of treatment have been completed. Patients will be randomized 1:1:1 in Group A receiving 0.4 g/kg/d of fish-oil lipid emulsion and 0.4 g/kg/d of a lipid emulsion mixture of ω-6 long-chain fatty acids (LCT) plus medium-chain fatty acids (MCT) (total dose of 0.8 g/kg/d of lipid emulsion); Group B receiving 0.8 g/kg/d of fish oil lipid emulsion and Group C receiving 0.8 g/kg/d of LCT/MCT emulsion.

The main objective is to determine whether 5 days administration of intravenous ω-3 fatty acid lipid emulsion is effective in normalizing interleukin-6 levels compared with LCT/MCT emulsions, and whether a 0.8 g/kg/d dose is more effective than 0.4 g/kg/d. Secondary outcomes include other inflammatory markers such as C-reactive protein, tumor necrosis factor alpha and interleukin-10, and parameters of morbidity, safety, nutrition and mortality.

Samples will be collected at the time when surgery is indicated and on days 0, 1, 3, 5 and 21 to determine inflammatory, nutritional, hepatic and safety parameters. In addition, clinical follow-up will be continued throughout the hospital admision and up to 1 year after surgery.

**Discussion:**

Studies of ω-3 fatty acids administered parenterally in esophagectomized patients are scarce. This study proposes to investigate the effect of combining fish-oil lipid emulsions administered parenterally with EN support. Potential benefits include fast incorporation of lipids to the cellular membranes and to the inflammatory cascade, and the use of only 1 pharmaconutrient.

**Trial Registration:**

FAR-NP-2017-01 EudraCT number: 2016-004978-17.

https://reec.aemps.es/reec/public/detail.html searching the EudraCT number.

**Version Identifier:**

Version 2, 08/06/2017.

## 1 Introduction

Esophageal cancer incidence increases with age, is more prevalent in men and is associated with risk factors such as smoking habit, obesity and alcoholism.^[1]^

This cancer is frequently associated with protein-energy malnutrition^[2]^ and usually has a poor prognosis due to late presentation and diagnosis, as well as patients not meeting criteria for curative treatment at the time of diagnosis. About 1 in 5 patients needs surgical resection.

Esophagectomy (EPG) is a major surgical intervention which involves total or partial esophageal resection and restoration of gastrointestinal continuity.^[1]^ Despite improvements in perioperative care, EPG continues to be associated with high rates of morbidity and mortality.^[1,2]^ In fact, post-operative complications are common, including both primary surgical events, such as anastomosis dehiscence or chylothorax, and adverse medical events, principally pneumonia.^[1,2]^

Nutritional support therapy is important in these patients due to the malnutrition associated with this pathology. Additionally, some enterally administered pharmaconutrients have been shown to be effective in the reduction of post-operative complications.^[2]^ Recent studies show that early enteral nutrition (EN) has other benefits: it increases immunocompetence, reduces infection rates and maintains intestinal functionality and structure. EN is cheaper and safer than parenteral nutrition, however post-operative EN is associated with tolerance problems such as: diarrhea, abdominal distension, and paralytic ileus or nausea, and only 50 percent of patients achieve their nutritional objective.^[3]^ Sometimes, symptoms become worse when caloric intake is increased, and EN discontinuation may be necessary.^[4]^

Omega-3 fatty acids, the principal components of which are eicosapenthaenoic acid and docosahexaenoic acid, are one of the pharmaconutrients with beneficial effects in post-operative morbidity. They are essential polyunsaturated fatty acids and fish oil derivates. Their administration can reduce proinflammatory cytokine production and regulate eicosanoid synthesis.^[5–7]^ Immunomodulation with ω-3 fatty acid based nutritional therapy has demonstrated benefits in reducing infectious post-operative complications and length of stay in elective gastrointestinal surgery patients, particularly in those who present with preoperative malnutrition.^[8,9]^ The enteral administration of ω-3 fatty acids in esophagectomized patients has been assessed in different studies.^[3,10–12]^ These assays verify the variability in the assimilation of ω-3 fatty acids when they are administered enterally, and show different results in relation to post-operative morbidity. However, 2 meta analyses^[13,14]^ conclude that enteral immunonutrition improves patient's morbidity without modifying their mortality.

The physiological role of ω-3 fatty acids is extensively reported, not only in maintaining nutritional homeostasis but also in the resolution of inflammatory mechanisms^[15]^ and in the course of inflammatory pathologies such as atherosclerosis or cancer. The effects of ω-3 fatty acid administration in the metabolic response are still being explored and discovered. Due to their protective effect in inflammatory processes such as colitis, ischemia-reperfusion syndrome, pain, sepsis or acute pulmonary pathology, the role of ω-3 fatty acids is relevant in artificial nutrition support.

PUFAs are precursors of proinflammatory as well as anti-inflammatory secondary messengers, and the physiological response produced depends on the balance of the lipid composition of the membrane. The eicosanoids derived from these polyunsaturated fatty acids present receptors in different kinds of cells executing different physiological functions. The study of these secondary messengers in the esophagectomized patient, may establish their correlation with the patient's clinical pathway depending on the capacity of the ω-3 fatty acids to modulate these secondary messengers.

Due to their iso-osmolarity, intravenous administration of lipid emulsions (LE) does not require a central line thereby avoiding any extra invasive treatment for the patient. In fact, LE may have protective properties for the vascular wall. Moreover, in patients considered for the study, the LE could be administered simultaneously with EN, so that gastrointestinal tract stimulation will not be interrupted, and the caloric goal achieved.

Studies of ω-3 fatty acids administered parenterally in esophagectomized patients are poorly reported in the literature. This study proposes to investigate the effect of combining fish-oil lipid emulsions administered parenterally with EN support in the framework of a randomized clinical trial. The difference between this approach and previous published studies is 2-fold:

- Parenteral administration facilitates fast incorporation of lipids to the cellular membranes, and consequently to the inflammatory cascade.^[16]^
- Use of only 1 parenteral pharmaconutrient (ω-3 fatty acids) facilitates the caloric goal and enables the study of the immunomodulatory effect of 1 nutrient without interference from other nutrients. When immunonutrition is administered enterally, the commercialized products available are combinations of different immunonutrients. Lipid emulsion comprising combined long-chain ω-6 long-chain fatty acids (LCT) and medium-chain fatty acids (MCT) is proposed as a control lipid to compare the effects on immunomodulation. These emulsions have the same physical appearance as ω-3 lipid emulsion. The LCT emulsions have an active role in the immunological system but are sensitive to peroxidation, whereas MCT emulsions do not participate in the immune response and are not sensitive to peroxidation, so in consequence the combination of both has no immunomodulatory effect.^[17]^

## 2 Methods

This trial is a prospective, single-center, randomized, double-blind study in patients diagnosed with esophageal cancer who post-EPG are treated with a lipid emulsion of ω-3 fatty acid or a mixture of ω-6 long-chain triglycerides/short-chain triglycerides 50%.

### 2.1 Study objectives

The main objective of this study is:

- To establish if the endovenous administration of ω-3 fatty-acid fish-oil lipid emulsions for 5 days in esophagectomized patients is effective in reducing inflammation – measured as seric concentration of interleukin-6 (IL-6) – compared to the administration of lipid emulsions of LCT/MCT. Additionally, to determine if doses of 0.8 g/kg/d are more effective than doses of 0.4 g/kg/d in the normalization of IL-6.

The secondary objectives aim to determine if the intravenous administration of ω-3 fatty-acid fish-oil lipid emulsions for 5 days in esophagectomized patients shows differences compared with the administration of LCT/MCT lipid emulsion with regard to:

- Seric concentration of other inflammatory parameters: C-reactive protein, tumoral necrosis factor-α (TNF-α), interleukin-10 (IL-10), interleukin-8 (IL-8) and soluble interleukin-2 receptor. In addition, in the follow-up, whether these markers, alone or in combination, show equal or greater efficacy of effect on the inflammatory response compared to IL-6.
- Post-operative complications: suture dehiscence, chylotorax, pneumonia and other infections.
- Safety, measured as hepatic impairment and alterations in coagulation parameters.
- Nutritional parameters: albumin, prealbumin and lymphocytes.
- Mortality: evaluated at hospital admission and at 1 year post-surgical intervention.

### 2.2 Study design

Prospective, unicentric, randomized, double-blinded study in patients with esophageal cancer and esophagectomized by the Ivor-Lewis or McEwan techniques. The study will be carried out in accordance with the good clinical practice in our hospital.

All participants will receive a lipid emulsion by continuous infusion in addition to EN support for 5 days post-surgery. The standard EN support will be initiated with Impact Neutre 500 ml (a normocaloric, hyperproteic and immunomodulatory diet composed of 3.9 g of lipids in each 100 ml, 1.8 g of MCT/100 ml, 0.61 g of ω-6 fatty acids/100 ml and 0.6 g of ω-3 fatty acids/100 ml, with a caloric input of 144 kcal/100 ml daily); it will be initiated at 21 ml/h and titrated to 1500 ml daily. When the body mass index (BMI) is greater than 25, the ideal adjusted body weight will be applied following the formula: “Ideal adjusted body weight = ideal body weight + (actual body weight - ideal body weight) ∗ 0.25 (if BMI 26–30) or ∗0.5 (if BMI > 30)”.

**Figure 1:**
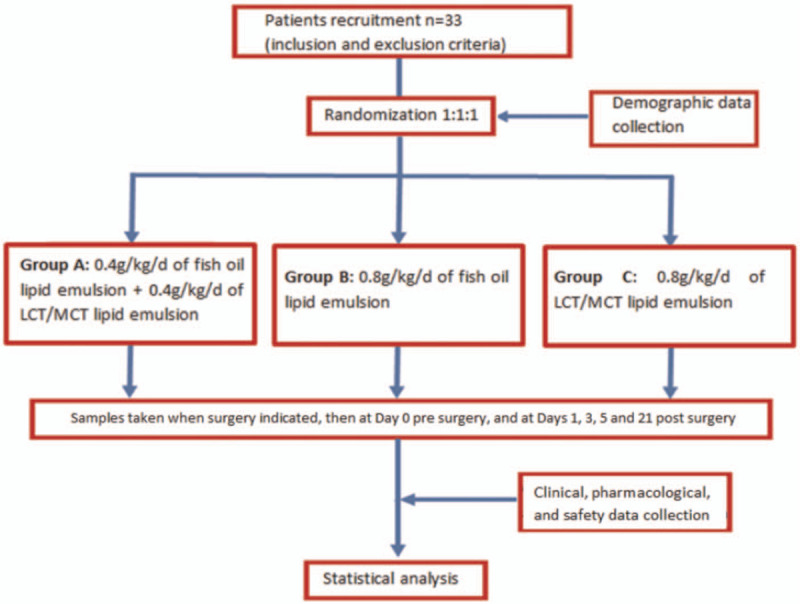
Study design flow chart.

Group A will receive a lipid emulsion mixture of 0.4 g/kg/d of fish-oil and 0.4 g/kg/d of LCT/MCT, Group B will receive 0.8 g/kg/d of fish-oil lipid emulsion and Group C will receive a lipid emulsion mixture of 0.8 g/kg/d of LCT plus MCT 50% (view at Fig. 1, which illustrates the diagram flowchart of this study)

**Figure 2:**
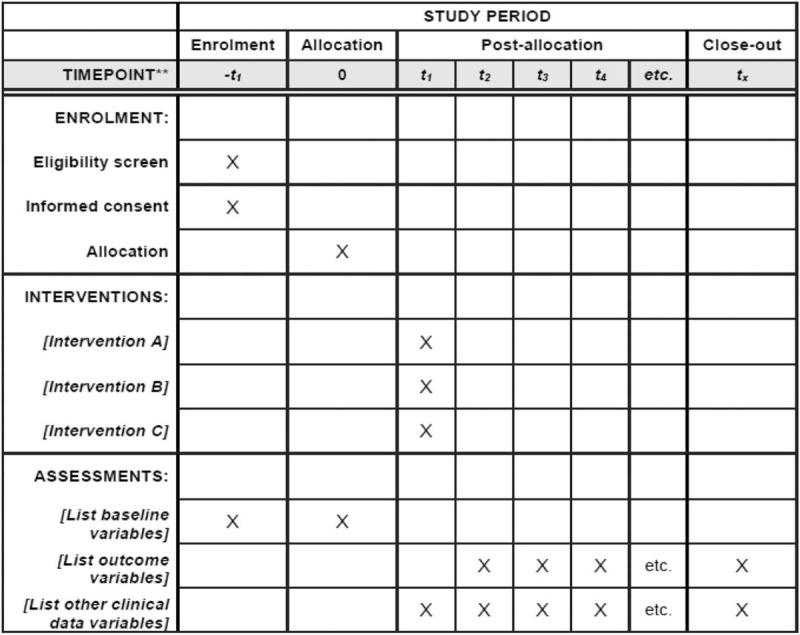
Standard Protocol Items: Recommendations for Interventional Trials (SPIRIT) flowchart.

This study will be conducted in accordance with Good Clinical Practice and this protocol follows the Standard Protocol Items: Recommendations for Interventional Trials (SPIRIT) checklist (Fig. 2 and Additional file 1, which shows the suitability of this study with the SPIRIT guidelines, Supplemental Digital Content).

#### 2.2.1 Study population

Patients diagnosed with esophageal cancer and candidates for EPG by the Ivor-Lewis or MacEwan techniques, and who meet the following **inclusion criteria**:

1. 18 years old or older, any gender and any race.
2. Willing and able to give their written informed consent (IC) for the study. If a subject is unable to give written IC, his legal representative may do so in his/her place.
3. Having access to the digestive tract.

Patients who meet the following **exclusion criteria** will not be chosen:

1. Hypersensitivity type 1 or idiosyncratic reactions to any of the lipid emulsion components.
2. Pregnant or breastfeeding women.
3. Plasmatic triglycerides concentration >3 mmol/L.
4. In receipt of chronic treatment with corticosteroids or immunosuppressants in the last month.
5. HIV diagnosis.
6. Transplanted.
7. Hepatic impairment classified as Child-Pugh grade B (significative functional compromise) or grade C (decompensated disease).

##### 2.2.1.1 Subject and/or treatment withdrawal criteria

Patients can voluntarily refuse to continue in the study at any time. In addition, presentation of any adverse effect related to the treatment will lead to a treatment withdrawal and to exclusion from the study.

All patients who present with surgical complications classifiable as grade IV of the Dindo-Clavien classification^[18]^ will be excluded from the study.

#### 2.2.2 Patients recruitment, randomization and allocation

All patients allocated for EPG following medical criteria, and those who meet the inclusion criteria will be candidates to participate in the trial.

The surgical team will be responsible for communicating the possibility of participating in this trial when IC is obtained for the surgical intervention. IC for participation in this trial will be collected before randomization.

Subjects will be randomized to 3 groups (1:1:1) by a pharmaceutical researcher after recruitment by the surgical team:

- Group A will receive 0.4 g/kg/d of fish-oil LE and 0.4 g/kg/d of a LE mixture LCT/MCT receiving a total dose of 0.8 g/kg/d of LE over 5 days post-surgery,
- Group B will receive 0.8 g/kg/d of fish-oil LE,
- And Group C will receive 0.8 g/kg/d of a LE mixture of LCT/MCT 50%.

This allocation will be blind for all patients and health personnel. The allocation sequence (computer-generated random numbers) will be generated by the pharmaceutical researcher and it will be performed by randomized blocks permutated with the block size defined at random. In the first step, blocks of size 3 with possible sequences ABC, ACB, CAB and CBA, and size 6 will be defined. In a second step the clusters will be randomized, and in a third step the sequences will be randomized within the blocks. This method ensures that the groups are balanced throughout the study, and that the researcher cannot predict the sequence. The pharmaceutical researcher will be responsible for patient assignation to the corresponding intervention group, and will also assign a numbered patient identification code for each patient in the trial. The composition of the emulsion administered to each patient will only be known by: the pharmaceutical researcher responsible for clinical trials, the pharmaceutical researcher who will validate its conduct, and the personnel who will conduct it.

#### 2.2.3 Number of participants subjects and trial duration:

Recruitment will last a total of 24 months from the approval date. Each subject will participate in the trial for a year post-surgical intervention.

On the basis of the results obtained and as referenced by Furukawa et al^[19]^ (a study of the values of IL6 in esophagectomized patients who received parenteral nutrition with ω-6 fatty acids or without lipids), and considering that the effect of administration of fish-oil emulsion (with ω-3 fatty acids) will be similar to the non-administration of ω-6 fatty acids, a sample size of 33 patients is estimated for 80% of potency and 5% of alfa error with 10% of anticipated losses.

### 2.3 Study variables

The main variable of this study is the variation of seric concentration levels of IL6 at randomization and on days 1, 3, 5, 21 post-surgery in esophagectomized patients receiving fish-oil lipid emulsions parenterally for 5 days compared with those receiving LCT/MCT emulsions.

The secondary variables will examine whether intravenous administration of fish-oil lipid emulsions instead of LCT/MCT for 5 days leads to differences from randomization and on days 1, 3, 5 and 21 post-randomization with respect to:

- Seric concentrations of inflammatory markers: RCP, TNF-α, IL-10, IL-8 and soluble interleukin-2 receptor.
- Morbidity measured as post-operative complications: suture dehiscence, chylotorax, pneumonia and other infections.
- Safety measured as hepatic impairment (total bilirubin, alanin-aminotranspherase, aspartate-amino transpherase, gammaglutamil transpherase, alkaline phosphatase) and alteration in coagulation parameters (INR, prothrombin time, platelet count).
- Nutritional parameters: albumin, prealbumin and lymphocytes.
- Mortality measured for a year after surgical intervention.

### 2.4 Treatments used in the trial

#### 2.4.1 Research drugs

The fish-oil emulsion employed in the study is commercialized as Omegaven, and the mixture emulsion of LCT/MCT is commercialized as Lipofundina

Lipids emulsions will be prepared at the Pharmacy Department with the same aseptic conditions used for NP (in a laminar flow cabinet). They will be delivered ready for administration in a multilayer bag, and will be stored and conserved in accordance with the normal procedures of the department of Pharmacy. The double blind will be facilitated by the identical galenic presentation of the lipid emulsions.

Emulsions will be dispensed when the trial recipe used in the University Hospital of Bellvitge is finalised.

#### 2.4.2 Others concomitant treatments

During this trial both the patient's usual medication and any support medication needed during admission will be maintained as usual.

### 2.5 Data collection

#### 2.5.1 Demographic, pharmacological, clinical and analytical data

Demographics, clinical and analytical data generated following normal clinical practice will be collected and recorded in the Data Collection Logbook.

Demographic data: age, gender, body weight, height and BMI will be recorded.

Pharmacological data: any adjuvant chemotherapy received by a patient after trial treatment will be recorded specifying the treatment scheme.

##### 2.5.1.1 Analytical data

- Inflammatory parameters: values of IL-6, RCP, TNF-α, IL-10, IL-8 y CD25 on days 0, 1, 3, 5 and 21 post-surgery will be recorded. These values have been selected according to what is reported in the literature in similar studies.^[19–25]^ Also, these same values will be recorded pre-operatively at the time surgical intervention is indicated to show the patient's baseline inflammatory status.
- Nutritional parameters: albumin, prealbumin and lymphocytes at the time surgical intervention is indicated and on days 0, 1, 3, 5 and 21.
- Hepatic parameters: total bilirubin, alanin-aminotranspherase, aspartat aminotranspherase, gammaglutamil transpherase and alkaline phosphatase at the time surgical intervention is indicated and on days 0, 1, 3, 5 and 21.
- Safety: INR, prothrombin time and platelet count at the time surgical intervention is indicated and on days 0, 1, 3, 5 and 21.

The total number of blood extractions per patient will be 6.

Clinical data: the following data will be recorded:

- Post-surgery complications such as chylothorax, dehiscence, pneumonia and other infections.
- Mortality: from the day of surgery to 1 year following surgery.
- Nutritional support: the amount of EN received by each patient will be recorded to determinate total calories administered.

##### 2.5.1.2 Quantification of cytokines

- Sample collection: Samples will be centrifugated at 700×*g* an hour post-extraction. Samples will be aliquoted and stored frozen at -80°C until analysis (1 year). Cytokine values will be determined by Enzyme-Linked ImmunoSorbent Assay (ELISA).

### 2.6 Response assessment

#### 2.6.1 Statistical analysis

Descriptive statistics will be employed for all collected variables data, such as baseline values for inflammatory, nutritional, hepatic and safety parameters, in frequency tables. For continuous variables, statistical descriptions will be used (n, average, standard deviation, value range and median), meanwhile grouped percentages will be used for categorical variables.

The main objective is to establish the difference between basal values of inflammatory parameters and values at day 5 post-randomization, and to assess differences between days 1, 3 and 21. The secondary objectives are to establish the difference between basal values of morbidity, safety and nutritional parameters and those values at day 5 post-randomization.

Significance will be calculated using Student's *t* test for matching data or Wilcoxon-test (non-parametric test) depending on whether normality can be assumed or not. To establish the differences among the 3 groups for the same period of time, Student's *t* test (parametric test) or Mann Whitney *U* test (non-parametric test) will be used, depending on whether normality can be assumed or not. For categorical variables, the chi-square test or the Fisher test will be used when necessary.

The significance level will be established with a 95% confidence interval (CI). Data will be processed with SPPS v 19.

### 2.7 Adverse events notification

All serious adverse events, regardless of their causal relation, will be reported to the study monitor immediately, sending a fax to +34932607884, in a maximum of 1 working day. According to articles 43 and 44 of the RD1090/2015, which regulates clinical trials in Spain, and in accordance also with sections 25 to 27 from the regulatory document concerning implementation of normal clinical trials (6th version, May 2008), the monitor will be responsible for communicating every serious and unexpected adverse event to the competent Health Authorities. (http://www.aemps.es/actividad/invClinica/ensayosClinicos.htm).

### 2.8 Monitoring

Monitoring tasks will be carried out by a monitoring company hired for this purpose. The person responsible for monitoring this study will have access to any clinical data required for the conduct of the trial and to all interim results. There will be 4 monitor-auditing visits that will take place at the beginning of the study prior to the first patient recruitment, when half of the patients are recruited, and when the follow-up of the last patient is finished, which means the end of the study. The monitor will be responsible for communicating any adverse events to the health authorities.

## 3 Discussion

These days, ω-3 fatty acids are playing an increasingly emergent role in artificial nutrition, due to the discovery of their protective and immunomodulatory effects in inflammatory processes.^[20–26]^

It is known that the PUFAs of cellular membranes are precursors of secondary messengers that can act as pro-inflammatory or as anti-inflammatory agents, and physiological response depends on the balance of the lipid composition of membranes.^[12,15,16]^

The study of these secondary messengers in esophagectomized patients, may establish their correlation with the patient's clinical progress, depending on the capacity of the ω-3 fatty acids to modulate these secondary messengers.

This study combines high-risk patients with considerable inflammatory response with intravenous administration of significant quantities of ω-3 fatty-acids, and may lead to the discovery of a new pharmacological strategy that is simple, safe and cost-effective. It will also provide useful data through the study of a new mediator.

## 4 Trial status

Protocol version 2 of 06/08/2017.

Patient recruitment began: 11/05/2018.

Approximate date of completed patient recruitment: 01/05/2022 (first approximate date was calculated on 01/05/2020, however it has suffered a delay because of COVID-19).

## Acknowledgments

We want to thank all the health personnel involved in the care of esophagectomized patients.

We thank CERCA Programme / Generalitat de Catalunya for institutional support

## Author contributions

MBT, JLT, ELB, and ASL conceived the work. ASL, MBT, ELB and JLT contributed equally to the writing of this study protocol. MFA, LFT, MMM, NVC, GCC, JBM, EPC, SNV contributed equally to the final review of this study protocol.

**Conceptualization:** Ana Suárez-Lledó, Josep M. Llop Talaveron, María B. Badia Tahull.

**Data curation:** Ana Suárez-Lledó.

**Formal analysis:** Ana Suárez-Lledó, Josep M. Llop Talaveron, Jordi Bas Minguet, María B. Badia Tahull.

**Investigation:** Ana Suárez-Lledó, Elisabet Leiva Badosa, Jordi Bas Minguet.

**Methodology:** Ana Suárez-Lledó, Elisabet Leiva Badosa, Josep M. Llop Talaveron, Leandre Farran Teixidor, Jordi Bas Minguet, María B. Badia Tahull.

**Project administration:** Elisabet Leiva Badosa, Mónica Fernández Alvarez, Leandre Farran Teixidor, Monica Miró Martín, María Núria Virgili Casas, Glòria Creus Costas, Jordi Bas Minguet, Sergio Navarro Velazquez, Elisabet Poyatos Canton, María B. Badia Tahull.

**Resources:** Mónica Fernández Alvarez, Leandre Farran Teixidor, Monica Miró Martín, María Núria Virgili Casas, Glòria Creus Costas, Jordi Bas Minguet, Sergio Navarro Velazquez, Elisabet Poyatos Canton, María B. Badia Tahull.

**Supervision:** Ana Suárez-Lledó, Elisabet Leiva Badosa, Josep M. Llop Talaveron, María B. Badia Tahull.

**Validation:** Ana Suárez-Lledó, María B. Badia Tahull.

**Writing – original draft:** Ana Suárez-Lledó.

**Writing – review & editing:** Ana Suárez-Lledó, Elisabet Leiva Badosa, Josep M. Llop Talaveron, Mónica Fernández Alvarez, Leandre Farran Teixidor, Monica Miró Martín, María Núria Virgili Casas, Glòria Creus Costas, Jordi Bas Minguet, Sergio Navarro Velazquez, Elisabet Poyatos Canton, María B. Badia Tahull.

## Supplementary Material

Supplemental Digital Content

## Supplementary Material

Supplemental Digital Content
