# Supplementary material for: Clinical, randomized, double blind clinical trial to study the effect of parenteral supplementation with fish oil emulsion in the nutritional support in esophagectomized patients
Source: Medicine (Baltimore). 2021 Jun 25;100(25):e26426. doi: 10.1097/MD.0000000000026426 (PMC8238298; doi:10.1097/MD.0000000000026426)
Supplement: Supplemental Digital Content [file medi-100-e26426-s001.pdf]

APPROVAL BY THE ETHICS COMMITTEE FOR INVESTIGATION WITH MEDICINAL PRODUCTS

Dr Enric Sospedra Martínez, Secretary of Ethics Committee for Investigation with Medicinal Products of Bellvitge University Hospital,

CERTIFIES

That the Ethics Committee for Investigation with Medicinal Products of the Bellvitge University Hospital, in the meeting of June 22nd 2017 (Act 12/17), after reviewing all the documentation presented by the Sponsor Dr. María B Badía Tahull from the Pharmacy Department at Hospital Universitari de Bellvitge, concerning the clinical trial with our ref. **AC012/17**, entitled:

"PILOT, RANDOMIZED, DOUBLE-BLIND TRIAL TO STUDY THE EFFECT OF PARENTERAL SUPPLEMENTATION WITH FISH OIL EMULSION IN THE NUTRITIONAL SUPPORT OF ESOPHAGECTOMIZED PATIENTS", code **FAR-NP-2017-01**, EudraCT number **2016-004978-17**.

Documents with versions:

Protocol	2nd version with data of 8th of June of 2017
Patien Information Sheet and Informed Consent	2nd version with data of 8th of June of 2017

And considers that:

- The procedure for obtaining informed consent (including the trial subject information sheets and informed consent forms mentioned above) and the planned subject recruitment are suitable and meet the requirements for obtaining the informed consent foreseen in Chapter II of Royal Decree 1090/2015.
- The planned compensation for participants is adequate, as well as the planned arrangements for compensation for damages that could be experienced by the subject.
- The planned procedure for personal data handling is adequate.
- The future use of biological samples obtained during the trial is adapted to Royal Decree 1716/2011.
- The sites and investigators specified in Annex II are considered adequate for the conduct of the trial, considering the suitability statements issued by the sponsor and the persons responsible for the relevant institutions.

After reviewing all the documentation presented, this Committee decided to issue a:

FAVORABLE OPINION

That in this meeting the requirements set down in current legislation (Royal Decree 1090/2015) were met in order for the decision of the aforementioned CEIm to be valid.

The CEIm, both in its membership and its procedures, complies with GCP guidelines (CPMP/ICH/135/95) and with current legislation governing its functioning and that the membership of the CEIm is that specified in Annex I.

That any member participating in the trial or declaring a conflict of interest has participated in the assessment or opinion on the application for authorization of the clinical trial.

Signed in L'Hospitalet de Llobregat, June 22nd 2017

Bellvitge Hospital
Comitè Ètic d'Investigació Clínica

Dr Enric Sospedra Martínez
Secretary
CEIm Hospital Universitari de Bellvitge

ANNEX I

MEMBERSHIP OF THE ETHICS COMMITTEE FOR INVESTIGATION WITH MEDICINAL PRODUCTS

**President**	Dr Francesc Esteve Urbano	MD - Intensive Care
**Vice president**	Dr Pilar Hereu Boher	MD - Clinical Pharmacology
**Secretary**	Dr Enric Sospedra Martínez	Pharmacist - Hospital Pharmacy
**Members-at-large**	Dr Jordi Adamuz Tomás	Nurse
	Dr María Berdasco Menéndez	Biologist
	Dr Enric Condom Mundo	MD - Pathology
	Dr Xavier Corbella Virós	MD – Internal Medicine
	Mrs. Consol Felip Farrás	Administrative
	Dr José Luis Ferreiro Gutiérrez	MD - Cardiology
	Dr Ana María Ferrer Artola	Pharmacist
	Dr Josep Ricard Frago Montanuy	MD - General and Digestive Surgery
	Dr Xavier Fulladosa Oliveras	MD - Nephrology
	Dr Margarita García Martín	MD - Medical Oncology
	Dr Josep Manel Llop Talaveron	Pharmacist - Hospital Pharmacy
	Mrs. Sonia López Ortega	Social Graduate
	Dr Sergio Morchón Ramos	MD - Preventive Medicine
	Dr Joan Josep Queralt Jiménez	Lawyer
	Dr Ricard Ramos Izquierdo	MD - Thoracic Surgery
	Dr Gemma Rodríguez Palomar	Pharmacist
	Dr Nuria Sala Serra	Biologist
	Dr Petru Cristian Simon	MD - Clinical Pharmacology

ANNEX II

SITES AND PRINCIPAL INVESTIGATORS PARTICIPATING IN SPAIN

AC012/17 PILOT, RANDOMIZED, DOUBLE-BLIND TRIAL TO STUDY THE EFFECT OF PARENTERAL SUPPLEMENTATION WITH FISH OIL EMULSION IN THE NUTRITIONAL SUPPORT OF ESOPHAGECTOMIZED PATIENTS, code **FAR-NP-2017-01,** EudraCT number **2016-004978-17.**

**Principal Investigator**	**Trial site**
Dra. María B Badía Tahull	Hospital Universitari de Bellvitge

Last updated date: 22/06/2017
