# Supplementary figures and images for: Clinical, randomized, double blind clinical trial to study the effect of parenteral supplementation with fish oil emulsion in the nutritional support in esophagectomized patients
Source: Medicine (Baltimore). 2021 Jun 25;100(25):e26426. doi: 10.1097/MD.0000000000026426 (PMC8238298; doi:10.1097/MD.0000000000026426)

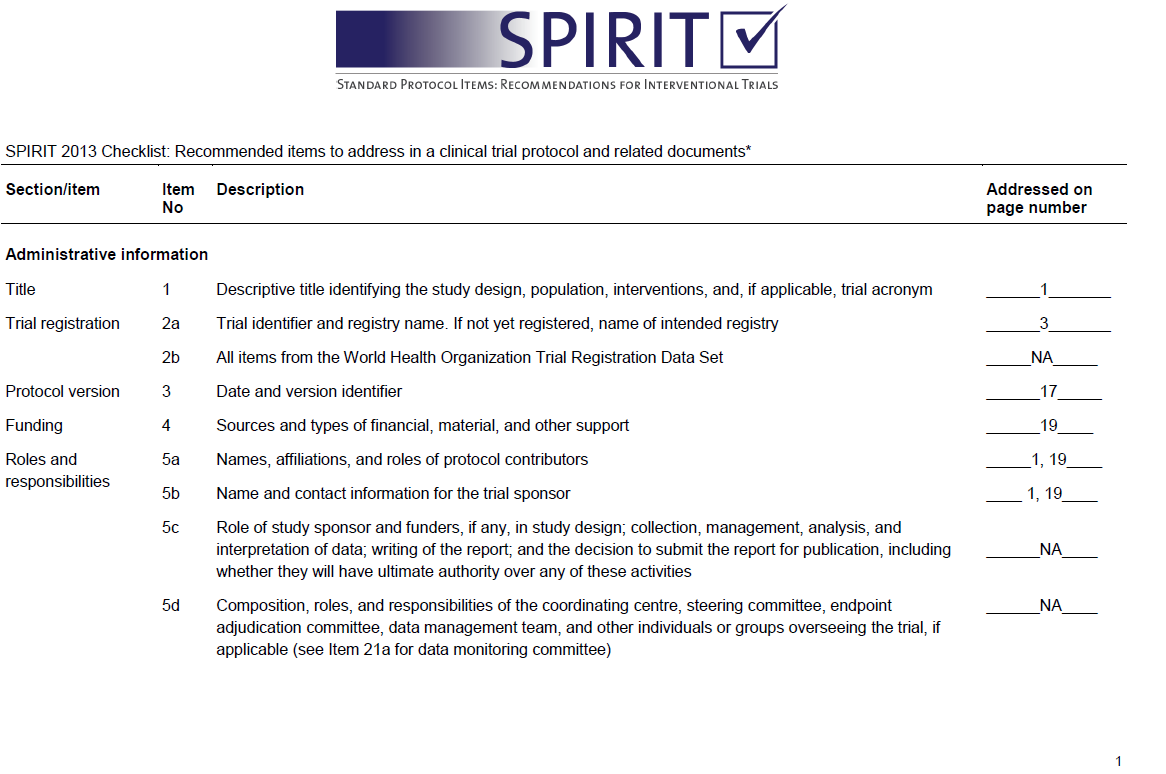


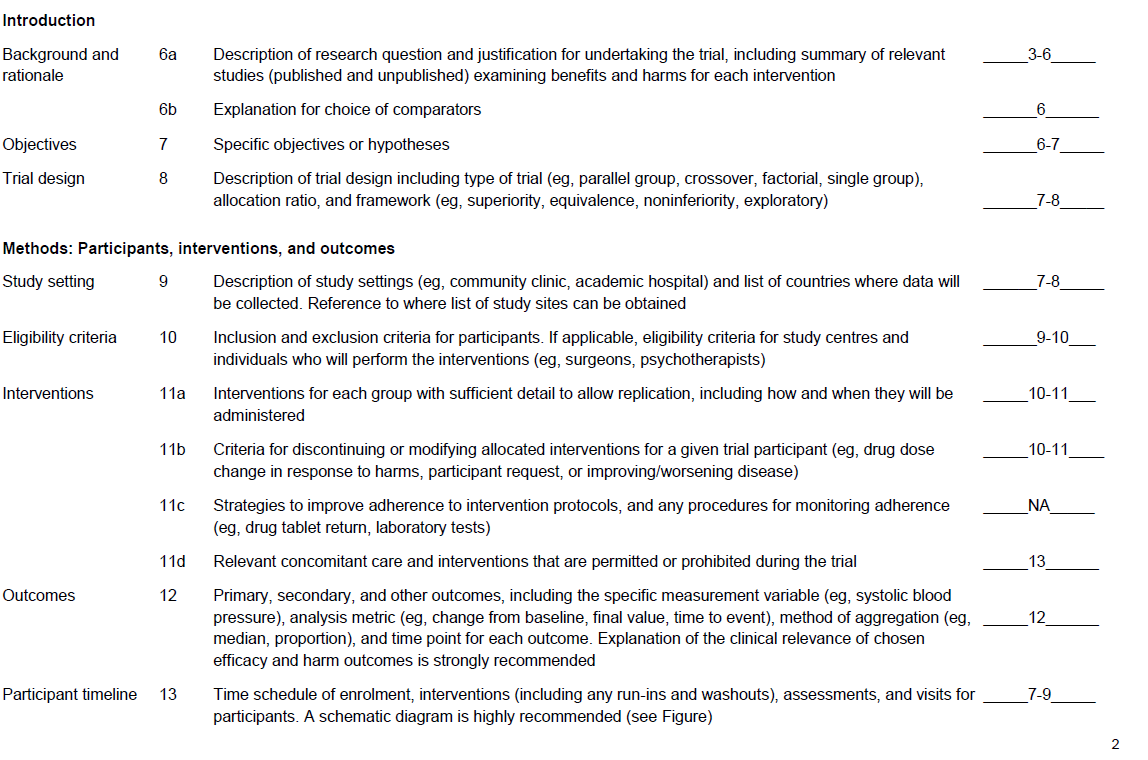


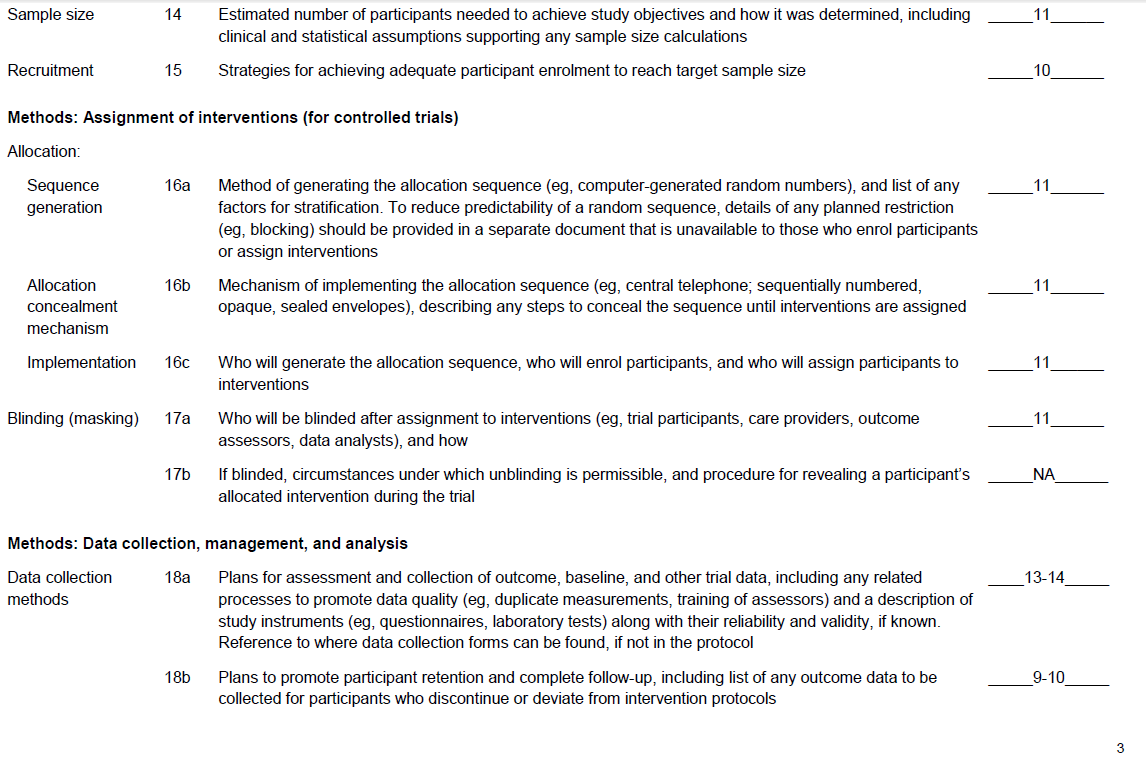


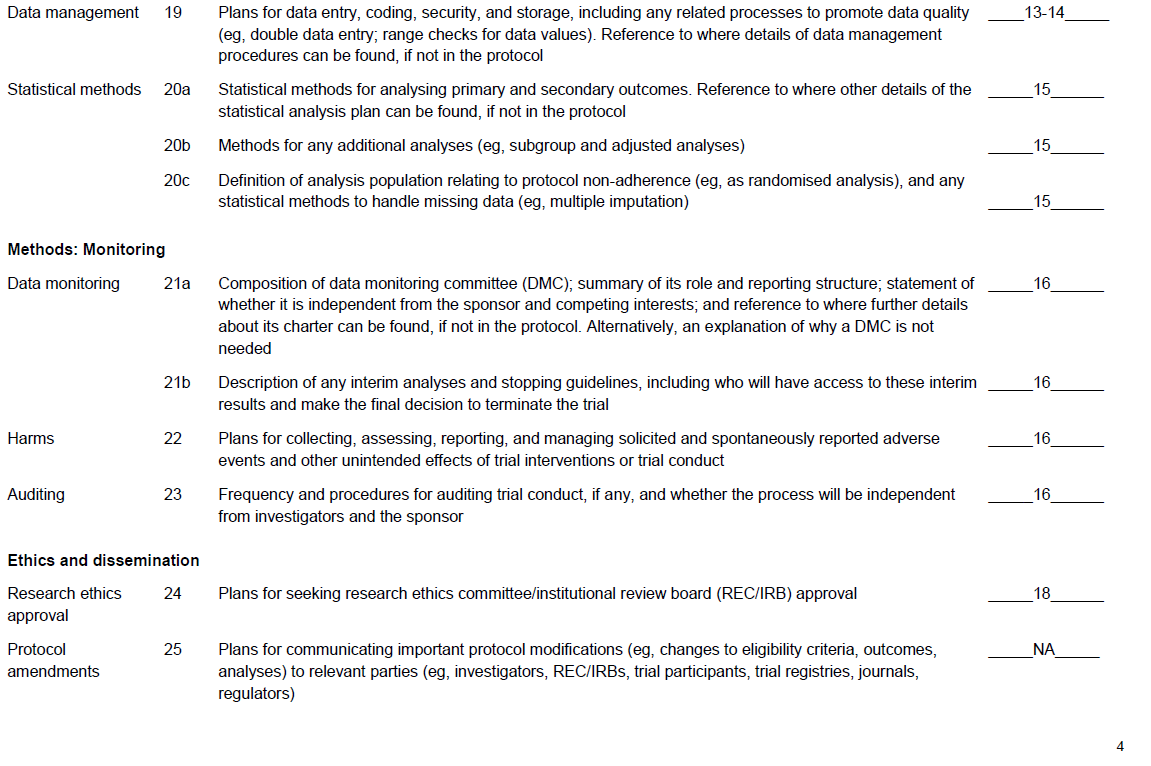


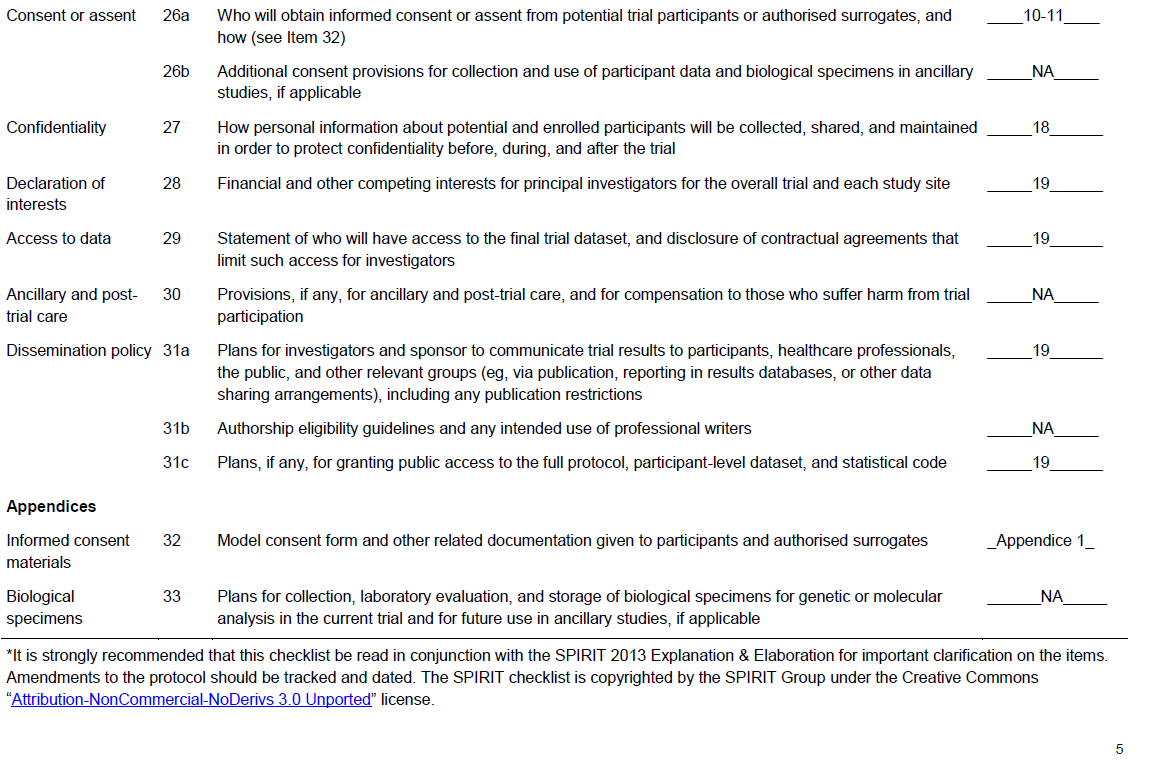

Supplement: Supplemental Digital Content [file medi-100-e26426-s002.docx]
